# The antimicrobial activity of theobromine against cariogenic microbes: an in vitro pilot study

**DOI:** 10.1038/s41405-024-00190-y

**Published:** 2024-02-01

**Authors:** Ismaeel H. Rafiq, Naile Dame-Teixeira, Thuy Do

**Affiliations:** University of Leeds, Leeds, United Kingdom

**Keywords:** Fluoridation, Oral diseases

## Abstract

**Objective:**

This pilot study aimed to compare the antimicrobial effect of theobromine, sodium fluoride, and a theobromine-sodium fluoride combination against the following caries-associated bacteria: *Streptococcus mutans* and *Actinomyces naeslundii*.

**Methodology:**

Antimicrobial susceptibility was tested via the broth microdilution method, with suspensions cultured on each microbe’s respective selective media. Shapiro–Wilk’s was completed and all the data showed normality (*p* > 0.05), and One-way ANOVA was applied to infer the significant differences in the viable counts between the groups.

**Results:**

All experimental conditions for both S. *mutans* and *A. naeslundii* groups resulted in a significantly lower bacterial abundance in comparison to the control medium, without any active antimicrobial agent (*p* < 0.001). There was no significant difference in viable count between the theobromine, fluoride, or combination groups against either microbe (*p* > 0.05).

**Conclusion:**

Theobromine’s antimicrobial activity against S. *mutans* and *A. naeslundii* was found similar to that of fluoride, whether used independently or in combination. Further testing of theobromine is necessary to assess its role as an alternative anticaries agent.

## Introduction

Caries is the most prevalent disease in the oral cavity [1] and results from microbiome dysbiosis on the biofilm covering tooth surfaces [2]. It can cause pain and discomfort, and significantly impact an individual’s quality of life and daily routine. As such, appropriate prevention against decay is of paramount importance within the dental field. Interestingly, natural substances have been suggested to modulate and maintain biofilm homeostasis. Also, in recent years, there has been an increase in popularity of oral hygiene products with natural ingredients [3]. Still, the research surrounding such agents is minimal and an appropriate natural alternative to modulate cariogenic biofilms has not yet been found.

Theobromine is a bitter alkaloid found mainly in cacao pods and preliminary studies suggest it can inhibit carious lesion formation with a mechanism of action parallel to that of fluoride. The use of theobromine as a dental agent is a novel concept therefore only limited research has been completed. An in vitro study on enamel blocks found that a theobromine-fluoride combination solution was more effective than fluoride alone in increasing enamel microhardness [4]. The clinical relevance of this cannot be stated as the study used an unknown fluoride concentration. Still, it is clear that theobromine can lead to improvements in enamel microhardness.

Fluoride’s ability to decrease enamel demineralisation has been explained by the mineral’s incorporation into the enamel crystal leading to an increase in size. Similarly, within an in vivo animal study using rats, theobromine has been seen to increase hydroxyapatite crystal size [5]. There was no fluoride group within the study therefore comparisons between crystal sizes cannot be completed. An alternative study on enamel blocks further discovered that 1% theobromine decreased the rate of enamel demineralisation, but again there was no comparative fluoride group [6].

Studies of similar design have also assessed theobromine’s ability to remineralise hydroxyapatite. An in vitro study on caries-like enamel lesions found that theobromine increased enamel remineralisation rates equal to that of fluoride, however a fluoride concentration of 1100 ppm was used [7]. This concentration is lower than standard adult toothpastes which contain between 1350 and 1500 ppm fluoride. Regardless, the results were confirmed by an analogous in vitro study which used a standard adult toothpaste, widely commercialised [8]. The concentration was undisclosed but conventional dentifrices typically contain between 1350 and 1500 ppm fluoride.

Aside from cariostatic properties, an in situ double blind randomised trial, using dentine blocks set within orthodontic brackets, found that theobromine toothpastes demonstrate substantially more tubule occlusion than 1500 ppm fluoride toothpastes and achieves this in a shorter time [9]. Practically, this can lead to lowered dentine hypersensitivity and quicker outcomes.

Still, specific studies into the antimicrobial activity of theobromine are rare, however, the cacao bean husk (CBH) extract has historically shown antiplaque activity within in vitro, in vivo, and clinical studies [10]. The cocoa bean originates from the cocoa tree, known taxonomically as Theobroma cacao L., and the predominant constituents of the bean husks is the active agent theobromine [11]. Due to the limited availability of studies using theobromine, studies utilising the CBH are relevant. As a result, it is clear there is a lack of research into theobromine’s independent potential as an antimicrobial as opposed to its other properties. Only two recorded studies comparing antimicrobial activity between theobromine and fluoride have been undertaken. Lakshmi et al. [12] (found theobromine toothpaste to be a more effective antimicrobial when compared against fluoride toothpaste. Their study used low amounts of fluoride (500 ppm/450 ppm) and followed a qualitative agar-disc diffusion method, which is known for having limited reliability or validity [13]. As such, the comparisons drawn have reduced real life implications. Demir et al. [14] found a theobromine toothpaste to be equally antimicrobial when compared to a traditional fluoride toothpaste. It is worth noting this traditional toothpaste contained triclosan, a known antimicrobial [15], thus the conclusions reached can’t be used to directly compare fluoride and theobromine.

## Aim

Characterise the antimicrobial activity of theobromine against oral bacteria with potential cariogenic traits, using fluoride as a comparison.

## Materials and methods

### Material preparation

In accordance with the literature regarding the microbiota of caries, the following cariogenic strains were selected for this study: *Streptococcus mutans* UA159 and *Actinomyces naeslundii* NCTC 10301 [16]. Frozen stock cultures of these strains were obtained from the University of Leeds Oral Biology microbial culture collection. The chosen microbes were cultured in brain heart infusion (BHI) broth then transferred to BHI agar to revive from the stock. Purity of the strains were confirmed via Gram staining and colony morphology. Selective agar media was further chosen to subculture each microbe throughout the study: mitis-salivarius with bacitracin (MSB) agar for S. *mutans*, and Cadmium Sulfate-Fluoride-Acridine Trypticase (CFAT) agar for A. *naeslundi*. The protocol for the MSB and CFAT were followed as described by Olga et al. [17] and Zylber and Jordon [18] respectively.

Artificial saliva and basal media were also prepared following the protocol by Wong and Sissons [19] and used to simulate the oral environment. Preliminary trials were run at varying ratios of basal media:BHI:artificial saliva; the chosen quantities of basal media and artificial saliva selected for the experimental culture medium showed the greatest microbial growth in the preliminary trial. The proportions were therefore set to encourage S. *mutans* and A. *naeslundii* replication. Adequate basal media nutrition was supplied, and the oral environment simulation was maintained via the artificial saliva.

To create the solutions with the active agents, sodium fluoride (Sigma-Aldrich, >99% purity) or/and theobromine powder (Sigma-Aldrich, >98% purity) were added to distilled water and then autoclaved, to dissolve and sterilise. Fluoride was set at a concentration of 1450 ppm, as this is the standard concentration within adult toothpastes. Theobromine was set at a concentration of 300 mg/L as at this level the solution would be safe for consumption, if used as a dentifrice, for all age groups [20].

### Experimental setting

Both S. *mutans* and A. *naeslundii* were suspended separately within a BHI broth followed by incubation for 48 h in an anaerobic cabinet at 37 °C. Additional dilution with BHI was then completed to standardise each broth suspension to an optical density of 0.5, in comparison to fresh BHI, using a spectrophotometer. With a pipette, 50 μL of this microbial suspension, 400 μL artificial saliva and 300 μL basal media were added into well plates. Following this, four experimental conditions were created for each microbe (Table 1) as 250 μL of the following solutions were added to the well plates: group 1 sterile water; group 2 1450 ppm fluoride solution; group 3 300 mg/L theobromine; group 4 725 ppm fluoride and 150 mg/L theobromine solution. Group 1, containing no antimicrobial agent, acted as the control group. The well plates were then incubated for 24 h, anaerobically, at 37 °C.

**Table 1:** List of conditions tested.

Table 1	List of experimental conditions
Test group	Treatment
1	No agent (Control)
2	1450 ppm fluoride
3	300 mg/L theobromine
4	150 mg/L theobromine + 725 ppm fluoride

Following serial dilution, the final stock solutions were plated on their respective selective media and incubated anaerobically for 96 h at 37 °C. Viable count was undertaken and colony forming units per mL (CFU/mL) was calculated for each test group. All experiments were carried out in triplicate.

### Statistical analysis

Following appropriate log transformations, all data sets were statistically analysed (SPSS Inc 27.0, Chicago IBM) with the Shapiro–Wilk’s test: the results for all groups showed data normality. Appropriately, following Bonferroni correction, one-way ANOVA was used for statistical analysis of the variance of the results. Confidence intervals at a level of 95% were also completed.

## Results

**Fig. 1:**
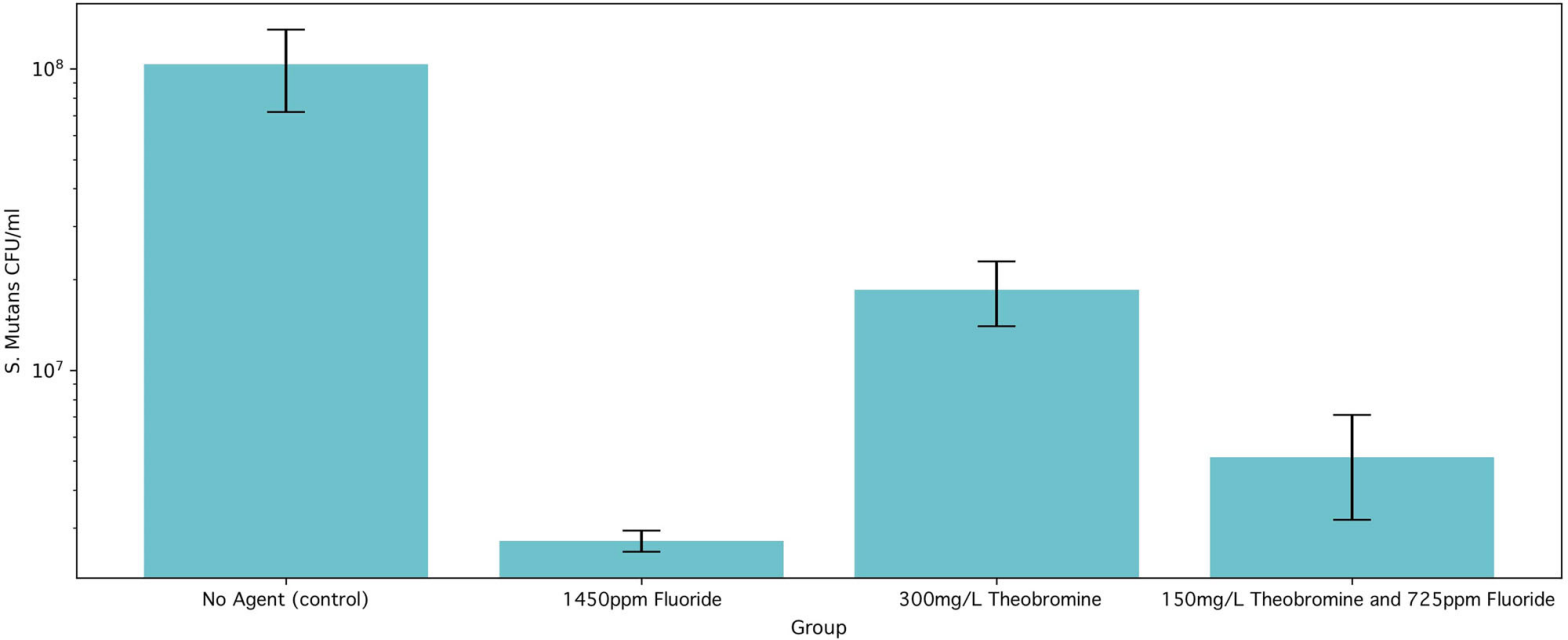
Bar chart displaying a logarithmic scale of bacterial growth. The amounts of viable bacteria are shown as colony forming units (CFU)/mL for S. *mutans*.

**Fig. 2:**
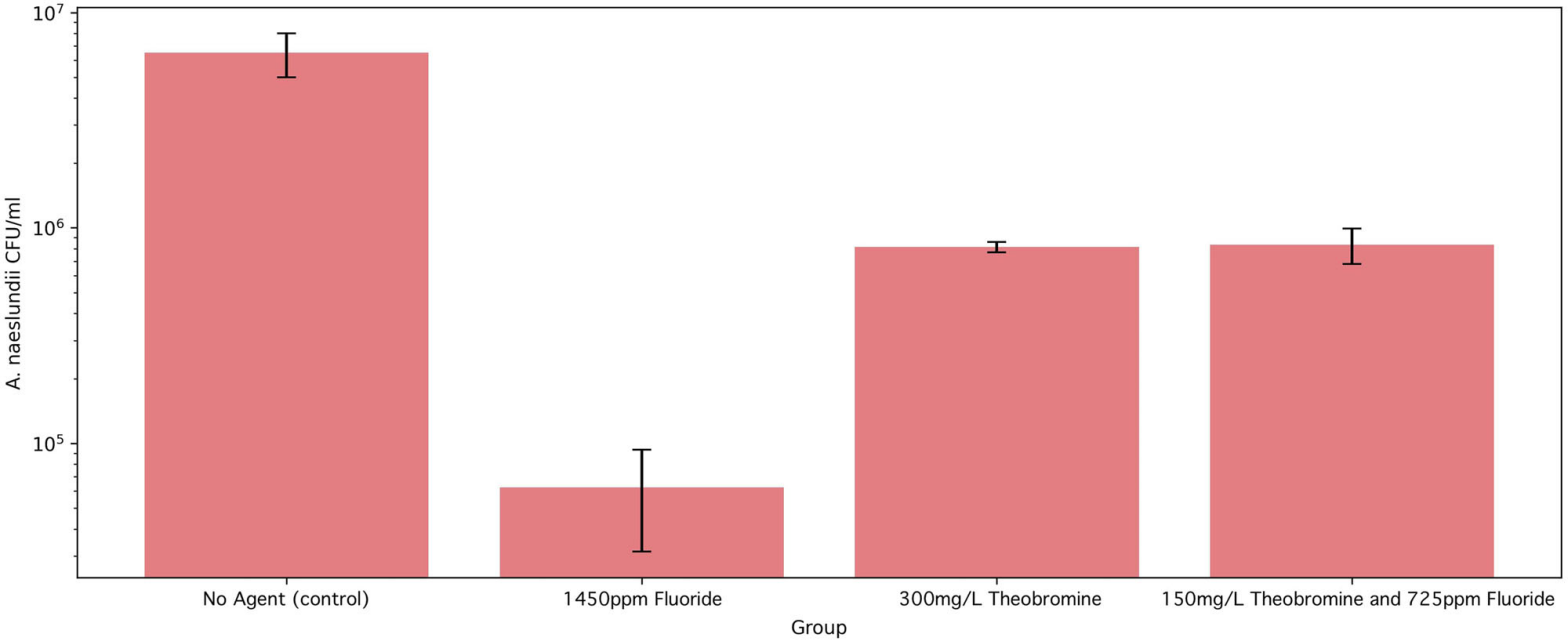
Bar chart displaying a logarithmic scale of bacterial growth. The amounts of viable bacteria are shown as colony forming units (CFU)/mL for A. *naeslundii*.

A clear trend in microbial abundance (CFU/mL) can be seen between the groups and all treatment conditions appear antimicrobial against both S. *mutans* (Fig. 1) and A. *naeslundii* (Fig. 2). The group with 1450 ppm fluoride (Group 2) resulted in the lowest mean microbial abundance for both S. *mutans* and A. *naeslundii* conditions. For the S. *mutans* cultures, there was no overlap in standard deviation between any group, and the theobromine-sodium fluoride combination (Group 4) showed the second lowest mean microbial abundance, followed by the theobromine-only group (Group 3) and then the control group (Group 1). However, for the A. *naeslundii* cultures, there was an overlap in standard deviation for Group 3 and Group 4 thus they both had a relatively equal decrease in microbial abundance in comparison to Group 1.

The groups with 1450 ppm fluoride (Group 2), with 300 mg/L Theobromine (Group 3) and with the 150 mg/L Theobromine – 725 ppm fluoride combination (Group 4) all showed a significantly lower bacterial abundance against both microbes when compared with the control group (Group 1) at a level of *p* < 0.001. However, there was no statistically significant difference in the bacterial abundance between the Groups 2, 3 and 4 conditions, against either microbe (*p* > 0.05).

## Discussion

The aim of this pilot study was to assess the antimicrobial activity of theobromine against oral cariogenic microbes, alone or in combination with fluoride. A protocol was developed to measure the viable count of S. *mutans* and A. *naeslundii* cultures post-incubation with different combinations of agents. It was found that 1450 ppm fluoride, 300 mg/L theobromine and a 150 mg/L theobromine – 725 ppm fluoride combination all demonstrated antimicrobial activity against both microbes. The data therefore confirms that theobromine has some antimicrobial potential against planktonic cells.

Within this in vitro study, methodology was adopted to replicate the oral environment. Artificial saliva was used within the culture medium to allow microbial growth rates to parallel clinical microbes. Oral cariogenic microbes were selected for this study from the literature to ensure antimicrobial measurements were obtained for relevant species. *S. mutans* in particular is known for its efficient adhesion, strong acid production and high tolerance to acidic conditions, which is why it’s often present within carious biofilms [21]. The validity of the results could have been increased with the use of a complete in vitro biofilm model. Intraorally, microbes aggregate into biofilms on the tooth surface; depending on biofilm physiology and exposure time, plaque or calculus can form. However, the current experiment primarily investigated the effects of the agents on planktonic bacteria cultured within broths. Future investigations can therefore utilise hydroxyapatite discs to mimic the tooth surface and allow biofilm growth. Nonetheless, the present study actively explored the general antimicrobial effects of the agents without specific biofilm testing.

Also, as a result of the study protocol, the antimicrobial agents were present throughout the 96-h incubation period. In reality, the dentifrice agent is washed away soon after brushing. This exposure was equal for all groups so fair comparisons can still be drawn, but future projects simulating the oral environment could consider alternative methods of agent application.

Additionally, only two cariogenic strains were investigated, and these were both cultured independently. Generalisability of the findings onto real world situations is therefore limited. The oral microbiome houses over 700 species which colonise the hard and soft tissues in the mouth [22]. Naturally, inside this ecosystem, communities of microbes interact within and across species, which hasn’t been replicated in the present study. The comparative influence of theobromine and fluoride on a greater range of species, including periodontal disease causing and health associated microbes, would have proved beneficial in understanding the complete antimicrobial properties of the agents. Utilising a mixed species culture also allows interactions between microbes to ensue. It is important to recognise S. *mutans* as the predominant cariogenic species [21] thus the current findings regarding the antimicrobial activity of the agents against S. *mutans* remains significant. Also, *Actinomyces* spp are common within carious root lesions therefore *A. naeslundii*’s selection for this study was appropriate [16].

Despite the sodium fluoride group having the lowest bacterial abundance for both microbes, there was no significant difference between the groups 2, 3 and 4 results. Reliability of a study can be increased by increasing the number of repeats, however, the narrow standard deviation shows the results were already clustered around the mean. Also, there is limited advantage to completing additional readings beyond the triplicate.

Regardless of the limitations surrounding validity and generalisability, this pilot study clearly demonstrates that pure theobromine has antimicrobial potential. This is in line with previous research as the CBH has already shown broad-spectrum antimicrobial properties [23]. Still, the findings of this current study challenge the conclusions drawn by Lakshmi et al. [12] who determined theobromine is a greater antimicrobial than fluoride. This is likely due to the higher concentrations of fluoride used within this present experiment. Alternatively, Demir et al. [14] found theobromine to be equally antimicrobial to fluoride within dentifrices, thus confirming the current findings. Still, within the body of research, this present study is the first to draw comparative data of antimicrobial activity between theobromine and fluoride alone, without the presence of uncontrolled additives.

It is also clear within the protocol that only a limited range of theobromine and fluoride concentrations were applied. Higher concentrations of theobromine should be tested and applied in combination with 1450 ppm fluoride, to form a more complete assessment of theobromine’s use as an adjunct. At a different concentration, both Shrimathi et al. [24] and Babu et al. [25] found a CBH mouthrinse to be as equally antimicrobial as a chlorhexidine mouthrinse against S. *mutans*. Chlorhexidine is already known to be stronger antimicrobial than fluoride against S. *mutans* [26] thus theobromine’s antimicrobial potential is apparent.

Antimicrobial adjuncts within dentifrices already exist. Triclosan has been added to various European oral hygiene products since 1985 and is a well-known antimicrobial agent [15]. Zinc also exhibits antimicrobial properties and within dentifrices has demonstrated significant reductions in the oral microbial population [27]. Due to the predominant use of fluoride alone, there are now concerns regarding the emergence of fluoride resistant microbial strains [28]. The use of an antimicrobial in combination with fluoride can therefore reduce the risk of developing microbial resistance. Unlike the alternative antimicrobial adjuncts, theobromine offers benefits beyond its antimicrobial activity. The need for such agents capable of modulating the microbial communities within dental biofilms is clear, and theobromine might be tested in order to observe weather it can to provide this action alongside other cariostatic functions. Similarly to fluoride, theobromine can also be added to dental materials. Initial testing indicates that theobromine additions to glass-ionomer cements leads to improved performance and caries preventative action, in the form of increased enamel microhardness and decreased biofilm formation on the restoration [29]. As such, a theobromine-containing restorative material could be helpful to reduce secondary caries rates. Future investigation is still necessary to compare the comprehensive anticaries action of theobromine in multiple forms, e.g. as a dental material, dentifrice or mouthrinse.

Irrespective of how theobromine is utilised, it is important to recognise its safety. Theobromine has likely already been consumed by the majority of the population as it is present within chocolate. It is readily absorbed and metabolised by the body, therefore safe to consume, however, at high consumption levels of up to 1.5 g of theobromine per day, symptoms, e.g. headaches, can develop [20]. This would require a considerate consumption of 300 mg/L theobromine solution to reach an unsafe dose. Still, to apply higher concentrations of theobromine in future studies, additional testing is needed on theobromine dosing. Also, further investigations are necessary to determine the agent’s biocompatibility with the oral mucosa, periodontal ligament and supporting cells. Detrimental effects of theobromine have thus far therefore not been explored. Instead, health benefits of theobromine consumption have been discovered and include reductions in glioblastoma prevalence as well as CBH ingestion preventing inflammatory gut damage [30, 31]. In comparison, the safety concerns of fluoride and current antimicrobial adjuncts, e.g., triclosan, are already known.

It is also worth mentioning the environmental impact of theobromine use. During cocoa production, only 10% weight of the cocoa fruit is used with the remaining 90% lost as waste or by-products [32]. The cocoa bean husk forms around 12% of the bean weight and is often discarded thus considered a significant agro-industrial residue [33]. Around 4.7 million tonnes of the cocoa bean are produced annually worldwide resulting in 700 thousand tonnes of CBH waste [34]. Recent bioconversion studies outline effective techniques for this CBH to be processed and for theobromine to be extracted [35]. As a result, the use of theobromine within dental products is a sustainable practice that will have a positive environmental outcome.

## Conclusion

In this in-vitro pilot study, a rigorous methodology was followed using standalone sodium fluoride and theobromine solutions, with no additional uncontrolled contents. According to the findings of this study, theobromine and sodium fluoride seem to exhibit equal antimicrobial action whether used independently or in combination. This data alone cannot be used as justification for the use of theobromine as a fluoride alternative, however the antimicrobial data will contribute to the field of research surrounding the potential use of theobromine as an anticaries agent.

Furthermore, within top of the range dentifrices, antimicrobial agents are already used in combination with fluoride. This study therefore provides comparative insight into fluoride and theobromine’s antimicrobial activity and highlights any potential antimicrobial benefit from using both agents in combination. Further work is indicated and should include a wider range of theobromine concentrations. Additional studies are needed to assess the clinical effectiveness of theobromine as an anticaries agent or as an adjunct to fluoride.

## Data Availability

The datasets used and/or analysed during the current study are available from the corresponding author.
